# Oncoplastie avec conservation mammaire dans le traitement du cancer du sein: à propos de 16 cas

**DOI:** 10.11604/pamj.2015.20.180.4897

**Published:** 2015-02-27

**Authors:** Wail Bouzoubaa, Meryam Laadioui, Sofia Jayi, Fatime Zahra Fdili Alaoui, Hakima Bouguern, Hikmat Chaara, Moulay Abdelilah Melhouf

**Affiliations:** 1 Service de Gynécologie Obstétrique Ii, CHU Hassan II, Fès, Maroc

**Keywords:** Cancer du sein, traitement conservateur, oncoplastie, breat cancer, Conservative treatment, oncoplasty

## Abstract

Le cancer du sein est actuellement le cancer le plus fréquent chez la femme, et pose un véritable problème diagnostique et thérapeutique. Le dépistage des lésions à un stade de plus en plus précoce, a permis une extension des indications du traitement conservateur radiochirurgical, qui était initialement limitées aux tumeurs de moins de 3 cm, unifocales, non inflammatoires. Par ailleurs, l'utilisation de traitements préopératoires permet d’étendre les indications du traitement conservateur à des tumeurs plus volumineuses. Parallèlement à cette extension des indications de conservation mammaire, on a observé le développement de nouvelles approches thérapeutiques notamment la chirurgie oncoplastique, technique du ganglion sentinelle et chirurgie stéréotaxique, dont les résultats initiaux sont très encouragent. A travers cette étude réalisée dans le service de gynécologie et obstétrique II du CHU HASSAN II de FES au MAROC, après l'analyse rétrospective de 16 patientes traitées par traitement conservateur et oncoplastie, nous avons voulus montrer notre aptitude a réalisé ses techniques chirurgicales et a bien prendre en charge ces patientes, mais aussi évaluer ces techniques en termes de résultat carcinologique et de résultat esthétique, aussi en terme de survie globale, survie sans métastase et en termes de récidive locale entre les plasties mammaires et les traitements usuels: mastectomie et traitement conservateur classique.

## Introduction

Le cancer du sein est le cancer le plus fréquent chez la femme en particulier dans les pays développés, plusieurs types et techniques chirurgicales peuvent être instaurés allant d'une tumoréctomie a une mastectomie. Le traitement conservateur du cancer du sein associe une chirurgie et au minimum une irradiation du sein. Chirurgicalement, différentes modalités sont bien connues et étudiées: les tumorectomies et les quadrantectomies. Celles-ci ont prouvé leur sureté carcinologique par rapport à la mastectomie. Depuis maintenant plus de dix ans, des techniques issues de la chirurgie plastique, sont utilisées pour diminuer les séquelles esthétiques des traitements conservateurs. Nous avons voulus, évaluer ces techniques de plasties mammaires en termes de résultat carcinologique et de résultat esthétique par l'analyse de 16 patientes ayant bénéficiées d'un traitement conservateur avec oncoplastie dans notre formation.

## Méthodes

Nous avons réalisés une étude rétrospective unicentrique incluant 16 patientes, opérées entre janvier 2009 et avril 2014, au centre hospitalier universitaire HASSAN II FES au Maroc, par traitement conservateur du cancer du sein, associant plastie mammaire et des traitements radio-chimiothéapeutiques (décidés en réunion multidisciplinaire).

## Résultats

L’âge moyen des patientes était 50,5 ans avec des extrêmes allant de 28 à 65 ans. Les caractéristiques de notre population, en termes d'antécédents carcinologiques étaient sans particularités, toutes les patientes présentaient pour la première fois un cancer du sein, la nulliparité a été retrouver chez 3 patientes soit 18,75%, une notion de prise de contraception oesto-progestatifs était retrouvée chez 13 de nos patientes soit 81,25%. 68,75% de nos patientes étaient ménopausées, et aucune n’était sous traitement hormonal substitutif. Dans 100% des cas, la tumeur était découverte par autopalpation d'un nodule du sein. Le sein gauche était atteint dans 75% des cas, 68,75% des tumeurs siégeaient au niveau du quadrant supéro-externe soit 11 de nos patientes, 12,5% siégeaient au niveau du quadrant supéro-interne, 6,25% au niveau de la jonction des quadrants internes et 6,25% au niveau de la jonction des quadrants supérieurs. La taille moyenne des seins des patientes opérées était un 95 bonnet C pour une taille moyenne tumorale évaluée cliniquement et radiologiquement à 2,68 cm (de 20 à 40 mm). Dans notre série, la répartition des tumeurs suivant leur taille montre une prédominance de tumeurs classées T2, soit 12 de nos 16 patientes: 75% des cas. L'atteinte ganglionnaire n'est retrouvée que dans 18,75% des cas. Toutes les patientes étaient M0. Sur le plan histologique toutes les patientes avaient un diagnostic avant la chirurgie et qui était un carcinome canalaire infiltrant chez 100% des cas. La chirurgie conservatrice du cancer du sein ne peut pas être proposée à toutes les patientes, associé à une plastie mammaire, ce traitement doit privilégier en premier lieu un résultat carcinologique satisfaisant. Le choix de la technique de plastie mammaire est guidé par plusieurs éléments: éléments liés aux patientes: volume mammaire, degré de ptose, diamètre de l'aréole, volume du sein controlatéral; Eléments liés à la tumeur: localisation, taille, type histologique.

Les indications actuelles des plasties mammaires dans le traitement conservateur du cancer du sein sont les tumeurs centrales superficielles, les tumeurs de plus de 3 cm et les tumeurs non accessibles à un traitement conservateur standard. Dans notre série les techniques utilisée faisaient appel a une technique en T inversé a pédicule inferieur dans 6,25% des cas, par une technique en J dans 6,25% des cas, par une incision radiaire interne en regard de la tumeur dans 18,75%, une incision en oméga dans 6,25% des cas, par technique externe dans 50%, et par une technique periaréolaire dans 12,5%. Les volumes de résections homolatéraux à la tumeur étaient de 200 g en moyenne. Un curage axillaire a été effectué dans 100% des cas. Dans 68,7% des cas, par la réalisation d'une incision directe du creux axillaire et dans 31,5% des cas par l'incision de plastie mammaire. La durée moyenne d'hospitalisation était de 3 jours avec des extrêmes de 2 à 5 jours. L’étude de nos marges de résection met en évidence que l'utilisation des techniques de plastie mammaire a permis d'obtenir des marges saines dans 87,5% des cas. Il existait une atteinte des berges dans 12,5% des cas soit 2 patientes sur 16 et qui ont été repris par une mastectomie. Nous avons eu 5 complications précoces (dans les deux mois postopératoires), soit 31,25%, type désunion de la cicatrice, lymphocèle et autres. Nous avons eu 17 complications tardives, et qui n’étaient pas très grave. L'asymétrie mammaire était la complication la plus fréquente. Dans notre série, la survie globale sans récidive à 5 ans est estimée à 85%, la survie sans métastase à 5 ans est estimée à 60%.

## Discussion

### Définitions et historique du traitement conservateur

#### Définition

Le traitement conservateur du cancer du sein associe une exérèse de la tumeur, le traitement des adénopathies axillaires et une irradiation du sein conservé. Il est actuellement le traitement de référence des cancers du sein T1 et T2 de petite taille, non inflammatoires. Ce traitement doit répondre à trois conditions: une survie identique à celle obtenue par la mastectomie, un risque de récidive locale faible, un résultat esthétique satisfaisant [1]. La chirurgie oncoplastique se définit comme l'utilisation de techniques de chirurgie plastique lors du traitement conservateur du cancer du sein. L'exérèse tumorale est corrélée à un geste plastique de comblement du défect glandulaire pour préserver la morphologie du sein et améliorer les résultats esthétiques [2].

#### Historique

Longtemps a prévalu le concept de Halstedt, qui considérait le cancer du sein comme une maladie locorégionale nécessitant une chirurgie d'exérèse large. Par la suite, le traitement s'est allégé, et la mastectomie simple avec curage axillaire est devenue le traitement de référence, parfois associée à une irradiation postopératoire. Puis, à la suite des travaux de Baclesse sur la radiosensibilité des cancers du sein et les résultats encourageants des premières séries de chirurgie conservatrice, s'est développé le concept de traitement conservateur. L’équivalence, en termes de survie, entre le traitement conservateur et la mastectomie est formellement démontrée pour les tumeurs jusqu’à 4cm [3] et 5cm [4]: deux études randomisées avec 20 ans de recul l'ont récemment confirmé. Une méta-analyse des 6 principaux essais thérapeutiques randomisant une association radiochirurgicale conservatrice contre une mastectomie a été publiée en 2005 par Jatoi. Plus de 4000 patientes ont été incluses avec un recul moyen de 14,7 ans. Elle a confirmé l'absence de différence significative sur la survie globale, mais une différence significative sur le risque de récidive locale en faveur de la mastectomie [5]. La méta-analyse de Yang parue en 2008 et portant sur 9 388 patientes retrouvait une survie globale identique à 3, 5, 10, 15 et 20 ans de recul. Le taux de récidive locale était augmenté dans le groupe traitement conservateur à 10 ans, cette différence n’était pas significative à 3, 5, 15 et 20 ans de recul [6].

#### Extension des indications du traitement conservateur

Classiquement, un traitement conservateur est proposé pour des tumeurs dont le diamètre est inférieur à 3 cm. Cependant, deux études randomisées [4] portant sur des lésions allant jusqu’à 5 cm ont montré que les taux de survie étaient identiques après traitement conservateur et mastectomie. La difficulté est alors d'ordre technique: il est le plus souvent impossible d'obtenir un résultat esthétique satisfaisant après tumorectomie large pour des lésions de cette taille, sauf en cas de sein très volumineux. En effet, une tumorectomie de très gros volume entraîne un risque élevé de déformation mammaire [7]. La chimiothérapie néoadjuvante [8] a permis aussi d’élargir les indications du traitement conservateur par la réduction de la taille tumorale.

### Nouvelles approches thérapeutiques

#### Ganglion sentinelle et cancer du sein

Le traitement conservateur du cancer du sein comporte le traitement des ganglions axillaires. Afin de diminuer la morbidité du curage axillaire classique, le concept de ganglion sentinelle a fait peu à peu son apparition. Il est fondé sur la diffusion progressive de l'atteinte ganglionnaire. Il consiste à injecter un traceur lymphophile (isotope ou colorant) au contact de la tumeur. La diffusion du traceur dans les lymphatiques du sein permet l'identification du ganglion «sentinelle», qui représente le premier ganglion drainant la tumeur mammaire et donc le premier relais lymphatique potentiellement métastatique. Dans une population de patientes atteintes d'un petit cancer du sein, dont le taux de risque d'atteinte ganglionnaire est de 30% environ, l'intérêt premier de cette technique est d’éviter un curage axillaire aux 70% des patientes dont l'aisselle est indemne. Dans des mains expérimentées, le taux de détection du ganglion sentinelle est actuellement de 95% [1]. Par ailleurs, on ne connaît pas la survie globale à long terme des patientes traitées par cette technique. Certains cas isolés de récidive axillaire après exérèse du ganglion sentinelle ont été décrits dans la littérature [9, 10].

### Chirurgie oncoplastique mammaire

#### Indications et contre-indications des gestes d'oncoplastie

Les indications sont réservées aux tumeurs palpables et foyers de microcalcifications dont le rapport taille tumorale ou extension radiologique par rapport au volume ou à la forme du sein laisse présager d'une séquelle esthétique de traitement conservateur (SETC). Les gestes d'oncoplastie sont également indiqués dans les localisations centrales aréolaires (lésions situées à moins de 2cm de l'aréole) et dans certains cas de reprise chirurgicale pour exérèse histologique incomplète ou étroite (après un premier traitement conservateur et avant radiothérapie) [2]. Le type histologique (carcinomes canalaires infiltrants, lobulaires et autres ou carcinomes canalaires in situ) n'intervient pas dans les indications d'oncoplastie, mais dans les indications de symétrisation dans le même temps opératoire. En effet, le risque de retrouver une multifocalité dans certains carcinomes lobulaires infiltrants fait recommander l'attente des résultats histologiques définitifs avant d'envisager une symétrisation. Les contre-indications sont [2]: deux tumeurs localisées dans des quadrants séparés du sein; microcalcifications occupant plus d'un quart du volume du sein; impossibilité de radiothérapie dans un délai de 2 mois: 1^er^ ou 2^ème^ trimestre de la grossesse, antécédent d'irradiation mammaire; certaines contre-indications relatives: maladie de système (intolérance à la radiothérapie); choix de la patiente.

### Différentes techniques « oncoplastiques » en fonction de la localisation tumorale

#### Tumeurs des quadrants inférieurs

##### Plasties mammaires en « T inversé »

**Figure 1:**
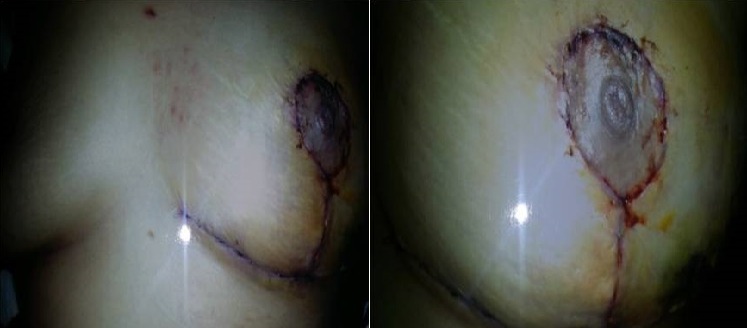
La rançon cicatricielle périaréolaire, verticale et dans le sillon sous-mammaire, en « T inversé » de l'une de nos patiente

La plaque aréolomamelonaire (PAM) est déplacée en haut après désépithélialisation d'un lambeau porte-mamelon à pédicule supérieur qui permet de transposer l'aréole de plusieurs centimètres si nécessaire. La glande mammaire est alors décollée du plan pectoral après incision dans le sillon sous-mammaire. Une large quadrantectomie emmenant au large la tumeur à l'union des quadrants inférieurs avec la peau en regard, puis les piliers glandulaires latéraux restants sont alors rapprochés et suturés l'un à l'autre pour reconstituer un nouveau volume glandulaire de forme normale. La symétrisation du sein controlatéral si nécessaire est effectuée avec la même technique et la même résection glandulaire. La rançon cicatricielle est donc périaréolaire, verticale et dans le sillon sous-mammaire, en «T inversé» (Figure 1).

##### Plasties mammaires verticales pures

Cette technique repose sur la même base que le «T inversé» sans la cicatrice du sillon sous-mammaire.

##### Plasties mammaires en «J» ou «L»

La verticale sous l'aréole est alors prolongée vers l'extérieur, elle permet de limiter la cicatrice du sillon sous-mammaire pour des seins plus volumineux.

### Tumeurs des quadrants externes

#### Plasties mammaires par technique externe

**Figure 2:**
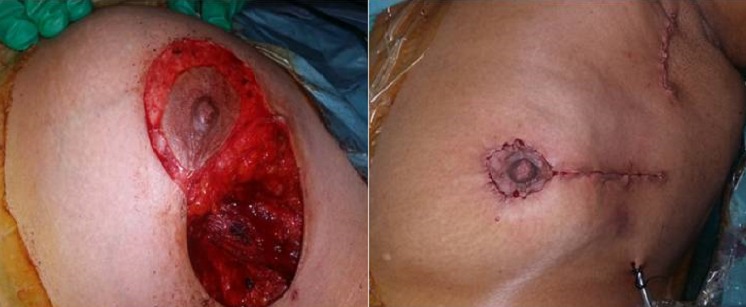
Incision radiaire externe en per opératoire et en fin d'acte de l'une de nos patients

On pratique une désépidermisation périaréolaire, sauf en regard de la tumeur. Une incision cutanée et glandulaire allant jusqu'au plan pectoral, puis une quadrantectomie emmenant la tumeur et la glande très largement en périphérie avec la peau en regard. Les piliers glandulaires restants inférieur et supérieur sont alors rapprochés et suturés l'un à l'autre (Figure 2). Le prélèvement axillaire peut être effectué par la même voie d'abord. La rançon cicatricielle est donc périaréolaire et radiaire externe. La symétrisation du sein opposé est en général effectuée avec la même technique (Figure 2).

#### Tumeurs des quadrants supérieurs

Ces localisations tumorales sont les plus difficiles à traiter car le segment II du sein (sus-aréolaire) est la zone du sein où la glande mammaire est la plus pauvre, surtout si la ptôse est importante. Plusieurs techniques opératoires sont adaptées pour ces tumeurs supérieures.

#### Plasties mammaires par technique périaréolaire ou «round-block»

**Figure 3:**
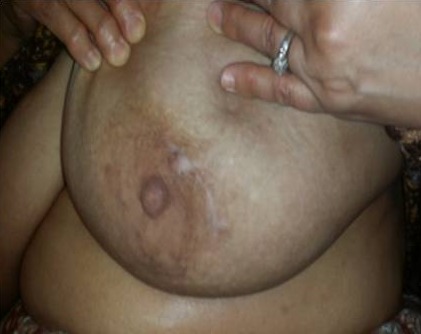
La rançon cicatricielle périaréolaire d'une plastie mammaire par technique périaréolaire ou « round-block »

Elle est utilisée pour les tumeurs à l'union des quadrants supérieurs, voire supéro-interne ou externe, mais assez proche de l'aréole. L'intervention débute par une désépidermisation périaréolaire plutôt ovalaire, sauf en regard de la tumeur si elle se trouve en face de cette zone. La peau est incisée à proximité du cercle externe, puis décollée au niveau des quadrants supérieurs et la glande est décollée du plan pectoral. Une tumorectomie large est effectuée plutôt en forme de «calisson d'Aix» afin de rapprocher plus facilement les piliers glandulaires. La rotation des piliers en dedans et en dehors permet de reconstruire le massif glandulaire. La rançon cicatricielle est donc uniquement périaréolaire (Figure 3). La symétrisation du sein opposé est en général effectuée avec la même technique opératoire.

#### Plasties mammaires en « T inversé » à pédicule inférieur

Cette technique est particulièrement adaptée pour les tumeurs à l'union des quadrants supérieurs. Le dessin préétabli reprend le tracé des techniques en «T inversé», le trou de serrure de la clé représente alors la zone de Tumorectomie. L'incision cutanée suit le tracé arrondi et va en monobloc jusqu'au plan pectoral. La peau est décollée latéralement le long des incisions verticales et horizontales inférieures. La glande est alors repoussée vers le haut après avoir été libérée latéralement. Elle reste perfusée par les artères perforantes profondes qui traversent le muscle pectoral et entre dans la glande mammaire. La symétrisation du sein opposé est en général effectuée avec une technique à pédicule supérieur plus simple à effectuer mais qui donne les mêmes résultats et les mêmes cicatrices.

#### Plasties mammaires par technique en «V» ou en «oméga»

Le dessin préopératoire est un V plus ou moins plat s'il est situé très au-dessus de l'aréole. En revanche, s'il est plus proche d'elle, il épouse la PAM à sa partie supérieure et prend donc une forme d'oméga. L'incision cutanée suit le tracé préétabli et la résection glandulaire est plutôt centrée sur la zone à réséquer. Il s'agit d'une résection monobloc cutanée et glandulaire allant jusqu'au plan pectoral emmenant largement la tumeur. Les quadrants inférieurs et la PAM sont ensuite ascensionnés et suturés à la partie supérieure de la tranche de section.

#### Tumeurs des quadrants internes

Souvent extrêmement difficiles à traiter, ces localisations vont pouvoir bénéficier de différentes techniques déjà décrites: pour les tumeurs supéro-internes, la plus adaptée est la technique en « oméga »; pour les tumeurs inféro-internes, les techniques verticales («T inversé» ou verticale pure) associées à un lambeau dermo-glandulaire désépidermisé de rotation pour combler le défect dû à la quadrantectomie; dans les autres cas, une incision radiaire interne en regard de la tumeur (emmenant la peau en regard) avec repositionnement de l'aréole en haut et en dehors.

### Tumeurs du sillon sous-mammaire

#### Plasties mammaires par technique du sillon sous-mammaire avec ou sans lambeaux glandulaires

Le dessin préopératoire passe 1 cm ou plus au-dessus et en dessous de la tumeur. L'incision cutanée suit ce tracé, la résection est large, jusqu'au plan pectoral emmenant la tumeur. On décolle ensuite la glande mammaire du plan pectoral, puis on abaisse l'ensemble jusqu'au sillon sous-mammaire.

#### Tumeurs centrales: « pamectomie »

Le problème dans les tumeurs centrales reste l'envahissement de l'aréole, très fréquent quand la tumeur a été proche de la PAM (moins de 2 cm de distance). Plusieurs techniques sont utilisées, en fonction de la taille et de la forme du sein. Le dessin préopératoire est périaréolaire, après l'incision cutanée, la peau est décollée en périphérie, la tumorectomie centrale monobloc emmenant la PAM va en profondeur jusqu'au plan pectoral. Ensuite la glande mammaire est décollée à sa face profonde et libérée du muscle pectoral. La reconstruction du volume glandulaire est effectuée par adossement des piliers glandulaires afin de reconstituer le cône glandulaire. Elle démarre de la profondeur vers la superficie afin de diminuer la base et d'augmenter la projection du sein reconstruit, la fermeture cutanée peut se faire en bourse ou de manière horizontale.

### Reconstruction de la plaque aréolo-mamelonaire

#### Mamelon

Pour le mamelon, on utilise une greffe du mamelon opposé s'il est assez volumineux, d'autres greffons peuvent être utilisés dont certains après tatouage tel que le greffon du lobule de l'oreille ou des petites lèvres. On peut également utiliser des lambeaux locaux.

#### Aréole

**Figure 4:**
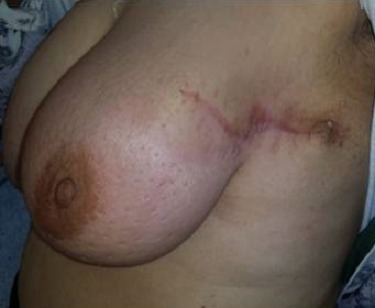
Une seule incision permettant de faire la tumoréctomie et le curage en même temps

L'aréole peut être également reconstruite soit par greffe, soit par tatouage. Dans notre série les techniques utilisée faisaient appel a une technique en T inversé a pédicule inferieur dans 6,25% des cas (Figure 1), par une technique en J dans 6,25% des cas, par une incision radiaire interne en regard de la tumeur dans 18,75%, une incision en oméga dans 6,25% des cas, par technique externe dans 50% (Figure 2), et par une technique periaréolaire dans 12,5% (Figure 3). Aucun cas de pamectomie n'a été réalisé. Les volumes de résections homolatéraux à la tumeur étaient de 200 g en moyenne. Un curage axillaire a été effectué dans 100% des cas. Dans 68,7% des cas, par la réalisation d'une incision directe du creux axillaire et dans 31,5% des cas par l'incision de plastie mammaire (Figure 4) ce qui rejoint les résultats de la littérature. La durée moyenne d'hospitalisation était de 3 jours avec des extrêmes de 2 à 5 jours.

#### Limites d'exérèse chirurgicale

L’état des berges d'exérèse correspond à la présence ou non de cellules carcinomateuses au contact des tranches de section chirurgicale, exprimée par la distance séparant les cellules tumorales des tranches de section chirurgicale. Une berge est dite saine si la marge mesurée au microscope est supérieure ou égale à 3 mm, et elle est considérée comme envahie si la marge mesurée au microscope est inferieure ou égale à 3 mm [(SOR 2001). Cela est variable selon les intervenants. Un des principaux intérêts du traitement conservateur par plastie mammaire est de permettre une résection tumorale avec des marges larges. Il a été démontré que des marges larges de résection sont associées à une diminution du risque de récidives, mais n'ont en revanche aucune influence sur la survie [11, 12]. Dans l’étude de Milan II, 708 patientes avec des tumeurs supérieures à 2,5 cm ont bénéficiées soit d'une tumorectomie (excision proche de la tumeur), soit d'une quadrantectomie (excision avec marges macroscopiques saines de 2-3 cm). Le taux de survie est identique dans les deux groupes, mais le taux de récidive locale à cinq ans est double dans le groupe tumorectomie [13]. Holland et al. proposent de réaliser des marges d'exérèse d'au moins 3 cm [14, 15] car ils ont réussies a démontrer que le nombre de foyers cancéreux situes a la périphérie de la tumeur primitive diminue progressivement a mesure que l'on s’éloigne des limites de celle-ci, les cellules malignes ont été retrouvées au niveau des marges dans 57% des cas si les marges sont de 1 cm et dans 17% si elles sont de 3 cm. L’étude de nos marges de résection met en évidence que l'utilisation des techniques de plastie mammaire a permis d'obtenir des marges saines dans 87,5% des cas, ce qui rejoint les résultats des différentes séries de la littérature [16]. Il existait une atteinte des berges dans 12,5% des cas soit 2 patientes sur 16 et qui ont été repris par une mastectomie car le remodelage glandulaire, induit lors de la réalisation de la plastie mammaire, fausse les repères du chirurgien ainsi le recours à une mastectomie est quasi impératif.

#### Complications de la chirurgie oncoplastique

Les complications du traitement conservateur avec oncoplastie peuvent être immédiates ou tardives (Tableau 1). Nous avons eu 5 complications précoces (dans les deux mois postopératoires) (Tableau 1), soit 31,25%. Ces complications ont nécessité une reprise chirurgicale dans 1 cas par le drainage d'un hématome. Les autres complications précoces rencontrées étaient: la désunion de cicatrice, et un cas de lymphocèle. Nous avons eu 17 complications tardives (au-delà de deux mois postopératoires) (Tableau 1), et qui n’étaient pas très grave. Certains patients avaient présentés 2 complications en même temps. L'asymétrie mammaire était la complication la plus fréquente. Les complications postopératoires ont été responsables d'un retard au traitement adjuvant (radiothérapie) dans 2 cas, soit 12,5% des cas et qui reste un pourcentage élevée par rapport aux autres séries de la littérature qui ne dépassent pas les 2%.

**Tableau 1:** Le nombre de complications postopératoires dans notre série par rapport à la série de STRAUB

COMPLICATIONS		Notre série	Série de STAUB(56)
**Immédiates**	Hématomes	1	10
	Abcès	0	3
	Nécrose cutanée	0	4
	Désunion de la cicatrice	3	2
	Lymphocèles	1	8
**Tardives**	Douleur	2	3
	Cicatrices rétractiles inesthétiques	4	6
	Asymétrie mammaire	8	5
	Sclérose cutanée	3	11

#### Facteurs de risque de récidive locale

Le risque de récidive existe après traitement conservateur. Mais sa survenue n'a pas d'influence sur le taux de survie à dix ans. La récidive locale constitue un échec du traitement et impose une nouvelle chirurgie avec souvent une mastectomie dans la mesure où le remodelage glandulaire de la plastie mammaire fausse les repères de la tumeur initiale. Ce risque de récidive locale persiste tout au long de la vie de la patiente. Dans tous les essais randomisés, le taux de récidive locale après traitement conservateur est compris entre 4 et 8% à cinq ans [17–19]. Trois facteurs de risque ont fait l'objet d'un consensus. Il s'agit de l’âge, de la présence d'emboles lymphatiques dans la tumeur et de l'atteinte des limites d'exérèse: âge: le risque de récidive mammaire diminue régulièrement à mesure que l’âge augmente [17, 20]. Ainsi, chez les femmes de moins de 35 ans, ce risque est de 29% environ à dix ans; il n'est plus que de 3% chez les femmes de plus de 55 ans; Emboles tumoraux: la présence d'emboles tumoraux intravasculaires; (sanguins ou lymphatiques) dans la tumeur est un facteur de risque de récidive locale: le risque est de 25% à dix ans, contre 8% lorsqu'il n'existe pas d'emboles [17, 21, 22]; atteinte des limites d'exérèse: une atteinte des limites d'exérèse (sur le mode infiltrant ou in situ) augmente le taux de risque de récidive locale [22, 23]. Ce taux est de 28% environ à dix ans si l'exérèse est incomplète, contre 8% lorsqu'elle est macroscopiquement large [23].

À l'inverse, certains facteurs font encore l'objet de controverses. Il s'agit du type histologique, du grade SBR, de la taille tumorale initiale [17, 24], de la présence de récepteurs hormonaux, de l'index de prolifération cellulaire et de la présence de carcinome intracanalaire en périphérie de lésions de carcinome infiltrant. Dans notre série le risque de récidive locale ne peut être étudié car la plus part de nos patientes sont toujours sous traitement adjuvent et n'ont pas encore atteint les 5 ans de survie avec leurs pathologie, néanmoins la patiente ayant un âge jeune de 28 ans, et les deux autres patientes avec des berges atteintes bénéficient d'une surveillance plus rapprochée, et jusqu’à ce jour aucune de nos patiente n'a présentée de récidive locale.

### Les résultats et les séquelles du traitement conservateur du cancer du sein avec plastie mammaire

#### Les résultats carcinologiques

Seul deux études ont été publiées étudiant les résultats carcinologiques du traitement conservateur du cancer du sein. Clough et al. en 1992 et Cothier-Savey et al. [13, 15] dans le service du Pr Barruch en 1996, ont montrés que le taux de récidives locales à cinq ans est de 8,5 à 9,4% et le taux de survie à cinq ans de 86% à 95,7%. Ces deux études ont des taux comparables à ceux retrouvés dans les traitements conservateurs standards [25], mettant en évidence la sureté carcinologique de cette pratique. Dans notre étude, la survie globale sans récidive à 5 ans est estimée à 85%, la survie sans métastase à 5 ans est estimée à 60%.

#### Les résultats et les séquelles esthétiques

Les résultats esthétiques du traitement conservateur classique sont imparfaits dans 20 à 30% des cas, nécessitant une reprise chirurgicale à distance [26] dans 5 à 10% des cas. Cela a été confirmé dans une série de l'European Institute of Oncology de Milan pour des patientes ayant eu un traitement conservateur sans recours à des techniques de chirurgie plastique. Les déformations les plus importantes ont été observées pour les tumeurs de localisation centrale ou inferieure. Les déformations les plus fréquemment retrouvées sont: brides rétractiles du creux axillaire, déformations en «bec d'aigle» dans les tumoréctomies des quadrants inferieurs, déféct glandulaire, asymétrie de forme et de volume. Des facteurs de risque de mauvais résultats esthétiques doivent être considères: obésité, hypertrophie mammaire, siège dans les quadrants inferieur ou central, le rapport volume tumorectomie/volume du sein élevé, radiothérapie. Les indications doivent être posées avec soins, il ne faut pas étendre à tout prix le traitement conservateur, car une mastectomie avec reconstruction mammaire immédiate ou différée peut donner de biens meilleurs résultats esthétiques qu'un traitement conservateur mal conduit.

Pour essayer de traiter au mieux les séquelles esthétiques et afin de clarifier les indications opératoires en fonction du type de déformation, ces déformations ont été classées en 5 grades: **Grade 1**: il s'agit de malformations très modérées, secondaires le plus souvent à un manque de remodelage glandulaire après traitement conservateur. Ces séquelles sont le plus souvent bien acceptées par les patientes. **Grade 2**: la déformation du sein traité par chirurgie et radiothérapie entraîne une diminution du volume et de la ptôse. Le sein conserve une forme « normale », mais il existe une asymétrie de volume qui nécessite un geste sur le sein opposé. **Grade 3**: il s'agit dans ce cas de la même déformation que dans les SETC de grade 2 avec une asymétrie au profit du sein non traité. Mais dans ce cas, le sein traité n'a pas une forme normale; cette déformation nécessite donc une plastie de remodelage afin d'améliorer sa forme. **Grade 4**: il s'agit du même type de déformation que pour les séquelles de grade 3, mais la déformation du sein traité est alors beaucoup plus marquée. Dans ces cas, le remodelage du sein est impossible car il manque une partie importante du volume Mammaire, le tout associé à une cicatrice rétractile et adhérente. **Grade 5**: il s'agit dans ce groupe des cas d'asymétrie majeure, avec des seins traités impossibles à mobiliser en raison d'une sclérose massive («sein de marbre»). Dans notre série une classification de nos patientes en fonction du grade de SETC (Séquelles Esthétique du Traitement Conservateur) a permis d'objectiver que 5 de nos patientes présentent des séquelles GRADE 1, et 3 présentent des séquelles GRADE 2, pour le reste des patientes on n'a pas encore assez de recul pour juger des séquelles esthétiques.

#### Survie après traitement conservateur

Plusieurs études ont démontré que les traitements conservateurs (suivis de radiothérapie) ont un taux de survie globale identique aux mastectomies pour les tumeurs de moins de 5 cm (84,6% a cinq ans pour les mastectomies et 82,3% pour les traitements conservateurs dans les CCI stades I et II, à dix ans la survie globale s’échelonne suivant les études entre 62 et 93,3%) [25]. De même pour la survie sans métastase, ou nous retrouvons des résultats équivalents dans les différentes études. Les résultats de notre série se rapprochent aux différentes études rapportés dans la littérature avec un taux de survie globale à 5 ans de 85% et un taux de survie sans métastase de 60% (Tableau 2).

**Tableau 2:** Les résultats de survie du traitement conservateur de notre série par rapport à d'autres séries

Auteurs	Stade tumoral	Survie globale (%)	Survie sans métastase	Survie sans récidive
FISHER	T1 et T2	62	50	90
JACOBSON	T1 et T2	77	72	82
SHIMAUSHI	T1 et T2	93,3	77,5	91,5
NOTRE SERIE	T2	85	60	100

## Conclusion

Le traitement conservateur du cancer du sein, a subi de profondes modifications depuis une dizaine d'années, par le développement de la chirurgie oncoplastique et plus récemment la chirurgie stéréotaxique dont les résultats esthétiques et carcinologiques sont très encourageant permettant de réduire les séquelles postopératoires physiques et psychologiques. Mais ces techniques doivent faire l'objet d'une stricte évaluation au sein d’équipes multidisciplinaires. Cependant, ces nouvelles approches thérapeutiques ne doivent pas nous faire oublier l'importance de l'obtention de limites d'exérèse saines dans la prévention des récidives locales. L'appréciation de ces limites et la taille des lésions tumorales permettent de prédire la présence d'une maladie résiduelle et son importance.
